# The Lysine Demethylase KDM5B Regulates Islet Function and Glucose Homeostasis

**DOI:** 10.1155/2019/5451038

**Published:** 2019-07-28

**Authors:** Marie Balslev Backe, Chunyu Jin, Luz Andreone, Aditya Sankar, Karl Agger, Kristian Helin, Andreas Nygaard Madsen, Steen Seier Poulsen, Madhusudhan Bysani, Karl Bacos, Charlotte Ling, Marcelo Javier Perone, Birgitte Holst, Thomas Mandrup-Poulsen

**Affiliations:** ^1^Immuno-endocrinology Laboratory, Department of Biomedical Sciences, University of Copenhagen, Denmark; ^2^Institute of Pharmacology, Department of Neuroscience and Pharmacology, University of Copenhagen, Denmark; ^3^Immuno-endocrinology, Diabetes & Metabolism Laboratory, Instituto de Investigaciones en Medicina Traslacional, Facultad de Ciencias Biomédicas, CONICET–Universidad Austral, Argentina; ^4^Biotech Research and Innovation Centre (BRIC), University of Copenhagen, Denmark; ^5^The Novo Nordisk Foundation Center for Stem Cell Biology, Denmark; ^6^Department of Biomedical Sciences, Faculty of Health Sciences, University of Copenhagen, Denmark; ^7^Unit for Epigenetics and Diabetes, Department of Clinical Sciences, Lund University, Scania University Hospital, Malmo, Sweden

## Abstract

**Aims:**

Posttranslational modifications of histones and transcription factors regulate gene expression and are implicated in beta-cell failure and diabetes. We have recently shown that preserving H3K27 and H3K4 methylation using the lysine demethylase inhibitor GSK-J4 reduces cytokine-induced destruction of beta-cells and improves beta-cell function. Here, we investigate the therapeutic potential of GSK-J4 to prevent diabetes development and examine the importance of H3K4 methylation for islet function.

**Materials and Methods:**

We used two mouse models of diabetes to investigate the therapeutic potential of GSK-J4. To clarify the importance of H3K4 methylation, we characterized a mouse strain with knockout (KO) of the H3K4 demethylase KDM5B.

**Results:**

GSK-J4 administration failed to prevent the development of experimental diabetes induced by multiple low-dose streptozotocin or adoptive transfer of splenocytes from acutely diabetic NOD to NODscid mice. KDM5B-KO mice were growth retarded with altered body composition, had low IGF-1 levels, and exhibited reduced insulin secretion. Interestingly, despite secreting less insulin, KDM5B-KO mice were able to maintain normoglycemia following oral glucose tolerance test, likely via improved insulin sensitivity, as suggested by insulin tolerance testing and phosphorylation of proteins belonging to the insulin signaling pathway. When challenged with high-fat diet, KDM5B-deficient mice displayed similar weight gain and insulin sensitivity as wild-type mice.

**Conclusion:**

Our results show a novel role of KDM5B in metabolism, as KDM5B-KO mice display growth retardation and improved insulin sensitivity.

## 1. Introduction

Diabetes mellitus encompasses a group of chronic metabolic disorders characterized by hyperglycemia. While type 1 diabetes (T1D) is characterized by absolute insulin deficiency, type 2 diabetes (T2D) is characterized by insulin secretion insufficient to compensate for insulin resistance [1], stressing the importance of dysfunctional beta-cells also in the pathogenesis of T2D. Although T1D and T2D are etiologically different disease entities, in both diseases, discrete genetic risk alleles are associated with functional gene variants that regulate gene expression with impacts on islet function and survival [2–4].

The central dogma describing the conversion of DNA to RNA to protein has become increasingly complex over the past decades owing to the discoveries of numerous posttranscriptional and posttranslational events modifying protein production and activity. Whereas histone lysine acetylation is typically associated with transcriptional activation, histone lysine methylation leads to transcriptional activation or silencing depending on which lysine residue is modified [5], a process regulated by lysine methyl transferases and lysine demethylases (KDMs) [6]. There are six different KDM families, KDMs 1 to 6, including 2-4 subtypes with diverse functions. Most KDMs have been implicated in embryonic development, fertility, cell differentiation, senescence, and oncogenesis. Of particular relevance to this study, KDM5B acts on di- and trimethylated H3K4 and interacts with gene silencing polycomb-group (PcG) proteins [7]. In contrast, the KDM6 family acts on di- and trimethylated H3K27, and KDM6A regulates HOX genes controlling somatic patterning during development, whereas KDM6B activates proinflammatory cytokine gene transcription [8].

We recently demonstrated that preserving H3K27 methylation marks using the lysine demethylase (KDM) inhibitor GSK-J4 protects beta-cells from cytokine-induced apoptosis in rodent and human islets, possibly via reduction of NFkB levels and ER stress signaling [9]. GSK-J4, which targets KDM6 demethylases with the highest potency, has cross-reactivity with the demethylase KDM5B, which catalyzes demethylation of lysine 4 on histone 3 tail (H3K4) [9, 10]. In contrast to methylation of H3K27, methylation of H3K4 is associated with transcriptional activation and is of particular importance for transcription of essential beta-cell genes such as *ins1/2*, *MafA*, and *Glut2* [11, 12]. Accordingly, glucose-stimulated insulin secretion (GSIS) in islets from mice with beta-cell-specific deletion of the H3K4 methyltransferase Set7/9 is impaired [13]. Consistent with these findings, we recently showed that GSK-J4 improved GSIS accompanied by an increased level of the transcriptionally activating H3K4 methylation mark [9], but we did not pinpoint the precise mechanism.

Previous studies have demonstrated that GSK-J4 reduces lipopolysaccharide-induced production of proinflammatory cytokines by macrophages [14] and improves experimental autoimmune encephalomyelitis by restraining inflammatory macrophage responses and inducing tolerogenic dendritic cells, the latter promoting Treg cell activity [14, 15], suggesting that GSK-J4 would ameliorate immune-mediated diabetes. In this study, we wished to expand on our earlier observations on the effect of GSK-J4 *in vitro* [9] and to explore the therapeutic potential of GSK-J4 *in vivo* by assessing whether administration of GSK-J4 protects mice from developing experimental diabetes. Furthermore, due to the specific functions of KDM5B in gene transcription and the importance of H3K4 methylation on islet secretory function as reviewed above, we wished to clarify the importance of H3K4 methylation in islet function using a KDM5B KO mouse.

## 2. Materials and Methods

### 2.1. Reagents

Two to five *μ*g/g of the inhibitor GSK-J4 (*N*-[2-(2-pyridinyl)-6-(1,2,4,5-tetrahydro-3*H*-3-benzazepin-3-yl)-4-pyrimidinyl]-beta-alanine ethyl ester) (Tocris, #4594) [14] or vehicle (300 *μ*l DMSO/ethanol/physiological sn) was administered i.p. three times a week during the entire experiment. A GSK-J4 stock solution of 100 mM was prepared in dimethyl sulfoxide (DMSO) to preserve stability. Before injection, the stock solution was diluted 1 : 10 with ethanol and brought to a final concentration of 300-750 *μ*g/ml in sterile physiological solution. The beta-cell-toxic compound streptozotocin (STZ) was administered i.p. (40 mg/kg) once a day for five consecutive days to induce diabetes. STZ was dissolved (6 mg/ml) in 0.1 M cold sodium citrate buffer, pH 4.5 immediately prior to injection.

### 2.2. Microarray Analysis

mRNA expression was analyzed in INS-1 832/13 cells in the presence or absence of the KDM inhibitor GSK-J4 (*n* = 7). Basic Affymetrix and Experimental Quality Analyses were performed using the Expression Console Software v1.1.2. Probe summarization, and Data Normalization was computed using the Robust Multiarray Analysis (RMA) and Expression Console Software v1.4.1.46 [16]. Gene set enrichment analysis (GSEA) [17] was applied to the expression array data using KEGG (Kyoto Encyclopedia of Genes and Genomes) pathways. Probes corresponding to transcripts were used and ranked according to the *t* statistics in a paired *t*-test. GSEA was run with the highest occurrence for genes with multiple probes and considered pathways with 1-500 transcripts.

### 2.3. Mouse Genetics, Breeding, Housing, and Interventions

To test whether *in vivo* administration of GSK-J4 protected beta-cells subjected to an inflammatory assault, we injected C57BL/6 mice (*n* = 26) with multiple low-doses of STZ on five consecutive days in the absence or presence of GSK-J4. The mice were divided in two groups. One group of mice (*n* = 15) received GSK-J4 (2-5 *μ*g/g; in a volume of 300 *μ*l i.p.) starting at day -1 relative to the start of the STZ challenge and then every other day until the end of the assay at day 17, while mice in the control group (*n* = 11) received an equal volume of vehicle. Body weight was measured. At the end of the experiment, pancreata from vehicle (*n* = 3) and GSK-J4-treated (*n* = 4) mice were dissected and saved for immunohistochemistry. For the evaluation of the *in vivo* effect of GSK-J4 in a model of autoimmune diabetes, splenocytes from overt diabetic NOD mice were adoptively transferred into female NODscid mice as described [18], and the diabetes incidence was determined in vehicle (*n* = 16) and GSK-J4-treated mice (*n* = 15). To explore the effect of GSK-J4 on insulin and glucose levels *in vivo*, wild-type unchallenged C57BL/6 mice were administered with GSK-J4 2 *μ*g/g (*n* = 6) or equal amount of vehicle (*n* = 4) every other day. Glycemia and insulin were measured on day 3 after the first GSK-J4 administration. Studies were approved by the Institutional Care and Use Committee FCEyN, University of Buenos Aires. NOD/LtJ, NOD.CB17-*Prkdcscid*/J (NOD*SCID*), and C57BL/6 mice breeders were originally purchased at The Jackson Laboratory and were housed at IBioBA-MPSP vivarium.

To investigate the role of KDM5B, we employed a constitutive whole-body KDM5B KO mouse, generated by Albert et al. [7]. The constitutive KO mouse was generated by crossing a conditional KDM5B (a.k.a. Jarid1b) [19] with *Cmv*-cre transgenic mice on the BALB/c background [20] to obtain heterozygous mice, which were further intercrossed to generate *Jarid1b* knockouts. Mice were kept on normal chow (*n* = 9) or a high-fat western diet (HFD) (21% of the calories from fat and 34% from sucrose) (*n* = 7) for 13 weeks.

Animal care and experimental procedures were carried out in accordance with the guidelines of the Institutional Care and Use Committee of FCEyN-Univ. of Buenos Aires. All animals were maintained, and experiments were carried out in accordance with the institutional guidelines and approved by the Animal Experiments Inspectorate in Denmark.

### 2.4. Histological Analysis

To investigate the levels of H3K4me3 in GSK-J4-administered mice, pancreata were dissected, fixed in 10% phosphate-buffered formalin, and dehydrated prior to paraffin embedding. Seven *μ*m contiguous paraffin sections were cut on a rotating microtome (Leica). Insulin and H3K4me3 were detected by immunofluorescence staining. Briefly, antigen retrieval with citrate buffer (pH = 6) was performed on tissue sections; thereafter, sections were permeabilized with 0.3% Triton, blocked with 1% BSA, and incubated overnight with the following primary antibodies: monoclonal mouse anti-insulin (1 : 200; clone HB125) and polyclonal rabbit anti-H3K4me3 (1 : 200; ABCAM). Cell nuclei were stained with DAPI (Invitrogen). The antigens were visualized using Alexa Fluor 647 goat anti-rabbit or Alexa Fluor 488 goat anti-mouse (1 : 200). All images were digitally acquired with the same parameters and were not further processed. The staining intensity of insulin and H3K4me3 signal in insulin-positive cells (average fluorescence intensity of insulin- or H3K4me3-positive area) of at least 5 different islets in pancreatic sections from 3 to 4 animals per group was quantified by monochromatic thresholding using ImageJ image software.

For immunohistochemical analysis of pancreatic sections from the KDM5B-KO mice, dissected pancreata were fixed in 4% formaldehyde (VWR, Denmark). Paraformaldehyde-fixed pancreas tissue was embedded in paraffin and cut into five *μ*m sections using a microtome. Pancreatic tissue (one randomly selected representative section/pancreas) was coimmunostained with glucagon antiserum diluted 1 : 15,000 (ab4304 raised in rabbit) and insulin antiserum diluted 1 : 3,000 (HUI-018 nn raised in mouse). The kit Multivision Polymer Detection System from Thermo Scientific (cat. no. TL-012-MHRA) was used to visualize the presence of two proteins simultaneously. The sections were incubated with the primary antibodies overnight at 4°C, immunohistochemistry was performed using a biotinylated secondary antibody and a streptavidin biotin conjugate, and the reactions were visualized using diaminobenzidine. Sections were counterstained with hematoxylin. The relative area of immunoreactive cells was measured in six randomly selected islets per section in a blinded manner. All measurements were performed on images using the software Zen Black.

### 2.5. Islet Isolation

Pancreatic islets were isolated mice (*n* = 3‐4) by bile duct perfusion of the pancreas with Liberase (Roche, Hvidovre, Denmark). Digestion was stopped by the addition of HBSS buffer containing Mg^2+^ and Ca^2+^ (Gibco), 3 g/l BSA, and 0.5 g/l D-glucose and washed with HBSS three times. Islets were handpicked under a dissection microscope and cultured for 1 day in RPMI 1640 with GlutaMax (Gibco-BRL, DK), supplemented with 100 U/ml penicillin and 100 *μ*g/ml streptomycin, and 10% FCS (Gibco-BRL, DK).

### 2.6. Glucose-Stimulated Insulin Secretion

For each condition, 25 islets were transferred to medium containing 3.0 mM glucose for 3 hours to establish baseline insulin secretion. Next, 25 islets per condition were incubated for 30 min at 37°C in 3.0 mM Krebs-Ringer solution (pH 7.4, 0.1% BSA, 0.1 mM HEPES) followed by 30 min at 37°C in 17 mM Krebs-Ringer solution. Supernatants from both conditions were kept for insulin measurement. Insulin was measured using the Sensitive Insulin RIA kit (Linco Research).

### 2.7. Body Composition

Body weight was measured at indicated time points in young and aged female mice (*n* = 5‐8), and body composition was determined in unanesthetized mice at indicated ages by quantitative magnetic resonance imaging (MRI) using EchoMRI 4-in-1 (Echo Medical Systems, Houston, TX). Body length between nose and tail root was measured in anesthetized mice (*n* = 2‐4). Mice were sacrificed, and the right femur bone was excised. After carefully removing all adhering soft tissues, the length (mm) between the greater trochanter and medial condyle was measured (*n* = 5).

### 2.8. Metabolic Phenotype

Oral glucose tolerance tests (OGTT) were carried out in female mice at indicated ages. Mice were fasted for 16–18 hours with free access to water. Glucose (1.5 g/kg body weight) was administered by oral gavage. Concentration of blood glucose was measured at time points -30, 0, 15, 30, 60, and 120 min, using a glucometer (Ascensia Elite XL Diabetes Care System; Bayer HealthCare, Berkeley, CA). At time points -30 and 15 min, blood was collected from the orbital sinus for insulin measurement measured using the sensitive insulin mouse radioimmunoassay (RIA) kit (Linco Research). For insulin tolerance test (ITT), mice were fasted for 2 hours with free access to water. Mice were challenged with insulin i.p. (Actrapid; Novo Nordisk, Copenhagen, Denmark) at a dose of 0.75 U/kg. Blood glucose levels were monitored as in the OGTT.

### 2.9. Measurement of Growth Hormone (GH) and Insulin-Like Growth Factor-1 (IGF-1)

Plasma levels of GH and IGF-1 were measured in female mice (*n* = 4‐6) at indicated ages. After overnight fast, blood was collected from the orbital sinus and plasma was separated for the measurement of IGF-1 using the ACTIVE Mouse/Rat IGF-1 RIA kit (Diagnostic Systems Laboratories Inc.). Plasma GH was measured using the Rat/Mouse GH ELISA Kit (Millipore).

### 2.10. Western Blotting

Gastrocnemius or soleus muscles were dissected from mice (*n* = 4‐5) at age 37-42 weeks and homogenized in lysis buffer. The homogenate was centrifuged at 16,000 g for 20 min, and the supernatant was used for western blot analyses. Protein concentration in lysates from skeletal muscle was determined using the BCA protein kit (#23225, Pierce), and equal amount of proteins were diluted in NuPage LDS sample buffer (#NP0007, Life Technologies) containing 50 mM DTT and heated to 92°C for 5 min. 15 *μ*g protein per lane was separated on 4-12% Bis-tris gels (Life Technologies) and electroblotted to PVDF membranes (#88518, Thermo Scientific). The membranes were blocked for 30 min (#37543, Thermo Scientific) before incubating membrane with primary antibody diluted 1 : 1000 overnight at 4°C. Antibodies to phospho-GSK3beta (#9315), total GSK3beta (#9336), phospho-Akt (#9271), total Akt (#9272), GAPDH (#2118), and IRbeta (#3020) were obtained from Cell Signaling. The membranes were washed in PBS + 0.1% tween-20 and incubated for 1 hour at room temperature with a horseradish peroxidase- (HRP-) conjugated secondary antibody (anti-mouse IgG #7076 or anti-rabbit IgG #7074 from Cell Signaling). After three washes, the membranes were incubated for 5 min with chemiluminescent HRP substrate luminol (#WBKLS0500, Millipore) and visualized using the FlourChem E Imaging System (ProteinSimple, San Jose, CA). The bands were quantified using ImageJ.

### 2.11. Statistical Analysis

Results are presented as means + SEM. Statistical significance (*p* < 0.05) was determined using GraphPad Prism 6 software by one- or two-way ANOVA as indicated or Student's *t*-test for paired or unpaired comparisons of two conditions. For statistical analyses of microarray data, *t*-test and statistical analysis of microarray (SAM) analysis were performed to identify significantly differentially expressed genes between groups [21] using TMEV v4.0 software, and false discovery rate (FDR) was applied to account for multiple testing.

## 3. Results

### 3.1. GSK-J4 Does Not Prevent or Delay Hyperglycemia in Diabetes Mouse Models

**Figure 1:**
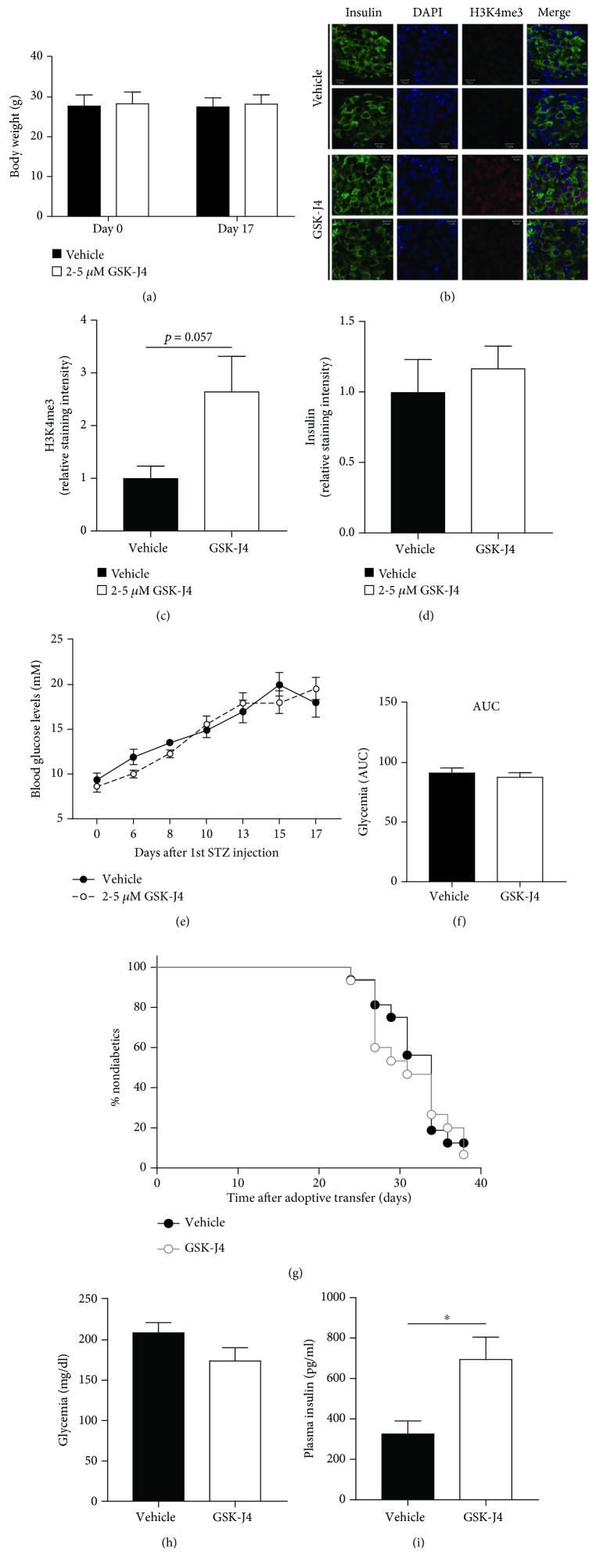
*In vivo* administration of GSK-J4. (a-f) Multiple low doses of streptozotocin (STZ) were employed to induce autoimmune diabetes in C57BL/6 mice (vehicle *n* = 11, GSK-J4 *n* = 15). (a) Body weight (g). (b) Representative images of pancreatic sections stained for insulin, DAPI, and H3K4me3 (*n* = 3‐4). Images are from two different mice. (c) Quantification of the H3K4me3 signal. (d) Quantification of the insulin signal. (e) Glycemic levels from day 0 to day 17. (f) AUC of glycemic levels. (g) Diabetes incidence as determined by hyperglycemia following adoptive transfer of diabetogenic splenocytes from overtly diabetic NOD mice into NODscid mice (vehicle *n* = 16, GSK-J4 *n* = 15). (h, i) Glycemia and insulin levels measured in C57BL/6 mice 3 days after 1st GSK-J4 (*n* = 6) or vehicle (*n* = 4) injection. Results are shown as means + SEMs. Statistical significance was determined using two-way ANOVA or unpaired *t*-test. ^∗^ *p* < 0.05.

We have previously shown that GSK-J4 protects rodent and human beta-cells from destruction caused by proinflammatory cytokines *in vitro* [9]. Therefore, we wished to address whether GSK-J4 administration might exert similar beneficial effects *in vivo* in the multiple low-dose STZ model of autoimmune diabetes. The mice tolerated GSK-J4 well, and there was no difference in body weight between groups (Figure 1(a)). GSK-J4 resulted in increased levels of H3K4me3 measured in pancreatic sections, indicating inhibition of KDM5B as intended (Figures 1(b) and 1(c), *p* = 0.057). GSK-J4 did however not inhibit or attenuate multiple low-dose STZ-induced hyperglycemia (Figures 1(e) and 1(f)).

Adoptive transfer of diabetogenic splenocytes from overtly diabetic NOD mice into NODscid mice is a more stringent model of autoimmune diabetes [22]. However, GSK-J4 administration did not have any effect on the time of onset or diabetes frequency when compared to control mice (Figure 1(g)). Taken together, these data demonstrate that *in vivo* GSK-J4 administration fails to inhibit immune-mediated diabetes development.

### 3.2. GSK-J4 Upregulates Gene Sets Comprising Olfactory and Glutamate Receptors

Being unable to prevent the development of STZ-induced or adoptive transfer of autoimmune diabetes by *in vivo* administration of GSK-J4, we pursued the previous observation of increased insulin secretion following GSK-J4 exposure *in vitro* [9]. To investigate whether we could reproduce our findings *in vivo*, we administered GSK-J4 to normal, unchallenged C57BL/6 mice and measured plasma insulin following three days. There was no change in glycemic levels (Figure 1(h)), but we were able to reproduce our *in vitro* findings and observed significantly increased levels of insulin following *in vivo* administration of GSK-J4 (Figure 1(i)). Pancreatic islet insulin staining was slightly but nonstatistically increased in GSK-J4-exposed mice (Figures 1(b) and 1(d)). To dissect the mechanism behind this observation, we applied gene set enrichment analysis (GSEA) on microarray data obtained from the beta-cell line INS-1 832/13 cells exposed to vehicle or GSK-J4 for 24 hours. After false discovery rate (FDR) correction of the microarray data, the GSEA yielded two significantly upregulated gene sets (*q* = 0.0001) containing genes involved in “olfactory transduction” and “neuroactive ligand-receptor interaction” (Supplementary Table 1). Genes contributing to the enrichment for the significant gene set “neuroactive ligand-receptor interaction” include genes encoding both ionotropic and metabotropic glutamate receptors.

### 3.3. KDM5B-KO Mice Have Enlarged Islets of Langerhans with an Increased Islet Insulin-Positive Area

Knowing that H3K4 methylation is important for the transcription of beta-cell genes and insulin secretion [12, 13], we considered it likely that the GSK-J4-induced improved insulin secretion was primarily achieved via KDM5B inhibition and thereby preservation of H3K4 methylation. To investigate the importance of H3K4 methylation on islet function, we employed a whole-body KDM5B-KO mice [7].

**Figure 2:**
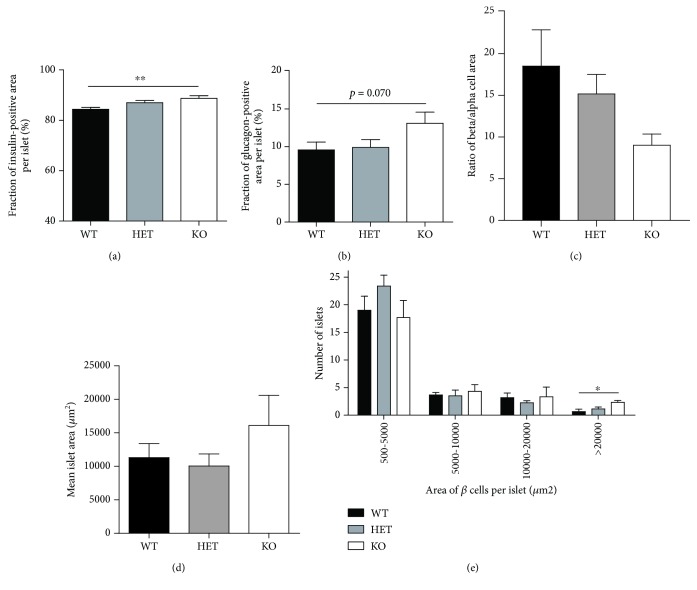
(a-d) The relative fraction of insulin- or glucagon-positive cells, ratio of *β*/*α* cells, and area of immunoreactive cells were measured in six randomly selected islets per section. (e) Quantification of area of all islets in each section using the software Zen Black. Results are shown as means + SEMs. Statistical significance was determined using one-way ANOVA. ^∗∗^ *p* < 0.01, ^∗^ *p* < 0.05.

We initiated our study by investigating islet morphology. Whole pancreata were dissected from KDM5B-KO mice, and immunohistochemical analyses were conducted on pancreatic sections. Islet morphology from KDM5B-KO mice resembled islets from wild-type (WT) mice (Supplementary Figure 1A). However, quantifying the area of randomly selected islets from the pancreatic sections showed that the insulin-positive area per islet from KDM5B-KO mice was significantly higher than from WT mice (*p* < 0.01) (Figure 2(a)), and likewise for the glucagon-positive area per islet, although not significantly (*p* = 0.070) (Figure 2(b)). The ratio of beta/alpha cell area showed no difference between WT and KO mice (Figure 2(c)). The islet area of KDM5B-KO mice tended to be larger than that of WT (Figure 2(d)), and when we examined the size distribution of the islets, KDM5B-KO mice had a significantly higher number of large islets (>20,000 *μ*m^2^) compared to WT mice (*p* < 0.05) (Figure 2(e)).

### 3.4. KDM5B-KO Mice Maintain Normoglycemia despite Impaired Insulin Secretion

**Figure 3:**
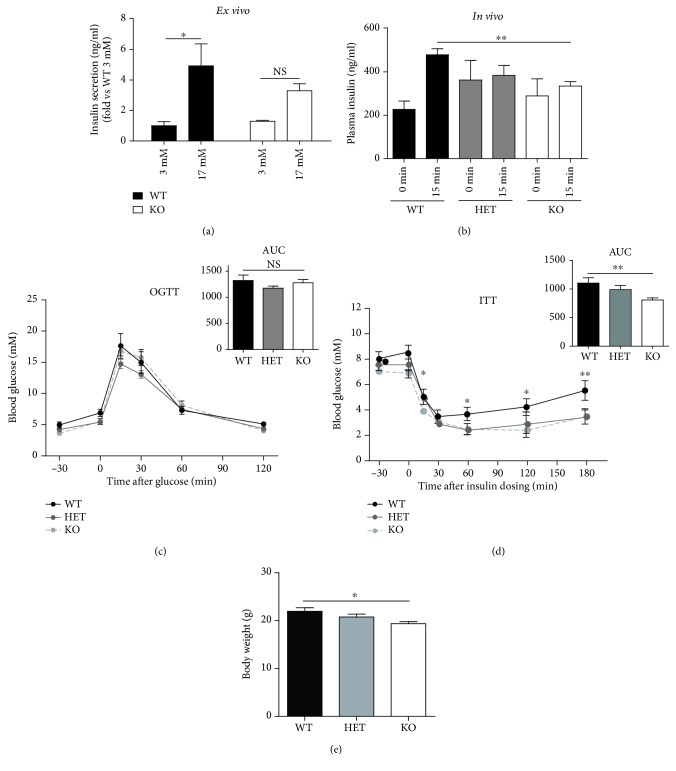
Phenotypic characterization of young KDM5B-KO mice. (a) Glucose-stimulated insulin secretion was measured *ex vivo* in isolated islets from WT and KO mice (*n* = 3‐4). (b, c) OGTT. Female mice (*n* = 5‐7) aged approximately 9 weeks were fasted overnight (16-18 hours) with access to water ad libitum. Blood samples were taken from a tail puncture at the indicated time points for measurement of glucose. (b) For measurement of insulin, a blood sample was taken from orbital sinus at time points -30 and 15 min for each genotype during OGTT. (c) Glucose excursion curves are shown for each genotype. AUC is shown for comparison of differences in glucose excursions. (d) ITT. Female mice (*n* = 3‐4) aged approximately 9 weeks were fasted 2 hours prior to ITT with access to water ad libitum. Insulin was injected i.p. at dosage 0.75 U/kg body weight. (e) Body weight (g) (*n* = 5‐8). Results are shown as means + SEMs. Statistical significance was determined by two-way ANOVA with Tukey's multiple comparisons test. ^∗∗^ *p* < 0.01, ^∗^ *p* < 0.05.

Having observed enlarged islets with an increased insulin-positive area per islet, we continued investigating our hypothesis that KO of KDM5B improved insulin secretion by maintaining higher levels of H3K4 methylation. Surprisingly, glucose-stimulated insulin secretion conducted on islets ex vivo revealed that KDM5B-KO mouse islets were incapable of significantly increasing their insulin secretion in response to high glucose in contrast to islets from WT mice (Figure 3(a)), despite similar levels of insulin content (Supplementary Figure 2A). Furthermore, following *in vivo* glucose challenge, young KDM5B-KO mice aged approximately 9 weeks secreted significantly less insulin compared to WT mice (*p* < 0.01) (Figure 3(b)). However, despite secreting less insulin, KDM5B-KO mice were able to maintain normoglycemia following an oral glucose tolerance test (OGTT), indicating improved insulin sensitivity (Figure 3(c)). Interestingly, insulin tolerance test (ITT) showed that recovery after hypoglycemia was delayed in KDM5B-KO mice (*p* < 0.05) (Figure 3(d)), supporting that KDM5B-KO mice maintain normoglycemia through improved insulin sensitivity.

### 3.5. KDM5B-KO Mice Display Reduced Growth

We next examined the effect of KDM5B-KO on body weight and composition by MRI on young mice of approximately 9 weeks of age. KDM5B-KO mice had a significantly lower body weight (*p* < 0.05) (Figure 3(e)). Absolute weight of lean mass was reduced (*p* < 0.01) (Supplementary Figure 2B), which following normalization to the body weight resulted in a relative lean mass similar to that of WT mice (Supplementary Figure 2C). The absolute weight of fat mass was similar between WT and KDM5B-KO mice (Supplementary Figure 2D), revealing a fat accumulation in KDM5B-KO mice (*p* < 0.05) following normalization to body weight (Supplementary Figure 2E).

**Figure 4:**
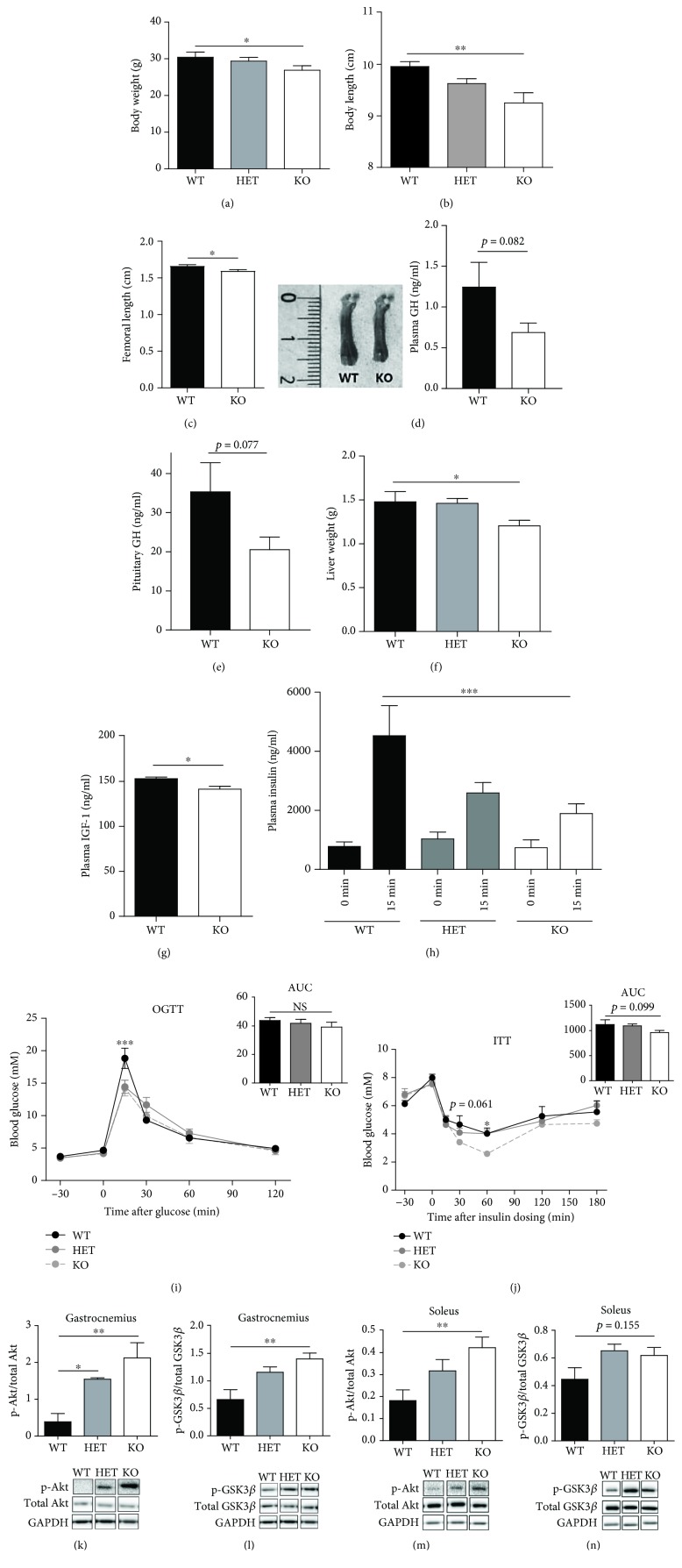
Phenotypic characterization of KDM5B-KO mice aged 30-35 weeks. (a) Body weight (g) (*n* = 5‐8). (b) Body length was measured between nose tip and tail root in anesthetized female mice (*n* = 2‐4) of 30-35 weeks of age. (c) Femoral length (cm) (*n* = 5) and representative image from a WT and a KO. (d) Plasma GH levels (*n* = 4‐6). (e) Pituitary GH levels (*n* = 3‐5). (f, g) Liver weight (*n* = 5‐8) and plasma IGF-1 levels (*n* = 4‐6) were measured in female mice of age 37-42 weeks. (h, i) OGTT. Female mice (*n* = 5‐8) aged 30-35 weeks were fasted overnight (16-18 hours) with access to water ad libitum. Blood samples were taken from a tail puncture at the indicated time points for measurement of glucose. (h) For measurement of insulin, a blood sample was taken from orbital sinus at time points -30 and 15 min for each genotype during OGTT. (i) Glucose excursion curves are shown for each genotype. AUC is shown for comparison of differences in glucose excursions. (j) ITT. Female mice (*n* = 5‐8) aged 30-35 weeks were fasted 2 hours prior to ITT with access to water ad libitum. Insulin was injected i.p. at dosage 0.75 U/kg body weight. (k-n) Protein expression levels of Akt and GSK-3*β* as determined by quantification of western blots on muscle samples from female mice (*n* = 4‐5) of age 37-42 weeks. Results are shown as means + SEMs. Statistical significance was determined by two-way ANOVA with Tukey's multiple comparison test. Results are shown as means + SEMs. Statistical significance was determined by unpaired *t*-test, one-way or two-way ANOVA. ^∗^ *p* < 0.05, ^∗∗^ *p* > 0.01, and ^∗∗∗^ *p* < 0.001.

With increasing age (from approximately 10 weeks of age to 32 weeks of age), percentage of body fat was normalized becoming similar to that of WT mice and likewise for percentage of lean mass (Supplementary Figures 3A-3B). Absolute fat mass was furthermore unchanged, while absolute lean mass correlated with the decreased body weight and was significantly reduced (*p* < 0.01) (Supplementary Figures 3C-3D). Distribution of gonadal, subcutaneous, and retroperitoneal fat was similar between WT and KDM5B-KO mice (Supplementary Figures 3E-3G). Interestingly, the mice aged 30-35 weeks maintained a reduced body weight (*p* < 0.05) (Figure 4(a)), which was accompanied by a significant reduction in body length (*p* < 0.01) and femoral bone length (*p* < 0.05) (Figures 4(b) and 4(c)) indicating a more general growth retardation. To investigate whether the impaired growth observed in KDM5B-KO mice was associated with impaired growth hormone (GH) action, we measured the levels of GH, insulin-like growth factor- (IGF-) 1, and liver weight in mice aged 30-35 weeks. GH levels tended to be reduced both in plasma and pituitary (*p* = 0.082, *p* = 0.077) (Figures 4(d) and 4(e)), and we observed a significant reduction in liver weight and levels of IGF-1 in plasma (*p* < 0.05) (Figures 4(f) and 4(g)). Taken together, these data show that lack of KDM5B leads to growth retardation.

### 3.6. Normoglycemia Observed in KDM5B-KO Mice Is Accompanied by Increased Insulin Signaling

While reduced somatic growth was observed in KDM5B-KO mice throughout life, we observed a difference in the percentage of fat mass between mice of 7-11 weeks and 30-35 weeks of age. We therefore wished to investigate the metabolic phenotype in KDM5B-KO mice aged 30-35 weeks to assess their glucose tolerance. Following oral glucose challenge, we saw an even more pronounced reduction in insulin secretion in KDM5B-KO aged 30-35 weeks compared to younger mice (*p* < 0.001) (Figure 4(h)). Despite the gross lack of insulin, the KDM5B-KO mice aged 30-35 weeks maintained normoglycemia following glucose challenge as did young mice (Figure 4(i)). ITT showed a delayed counter-regulatory response 30-60 min following insulin challenge, although AUC was not significantly reduced (*p* = 0.099) (Figure 4(j)). To elucidate the mechanism by which KDM5B-KO mice maintain normoglycemia despite secreting less insulin, we investigated the components of the insulin signaling pathway in skeletal muscle. The insulin receptor- (IR-) beta was unaffected by KDM5B-KO (Supplementary Figure 3H), but interestingly, phosphorylation of Akt and glycogen synthase kinase- (GSK-) 3beta was significantly increased in skeletal muscle from KDM5B-KO mice (Figures 4(k)–4(n)), further suggesting improved insulin sensitivity.

### 3.7. KDM5B-Deficient Mice Maintain Reduced Body Weight and Normoglycemia When Challenged with High-Fat Diet

Having found that KDM5B-KO improves glucose tolerance and insulin sensitivity when fed normal chow diet, we next wished to investigate whether KDM5B-KO might have the potential to improve glucose tolerance in a T2D model. To do this, we used the high-fat diet (HFD) model causing impaired glucose tolerance and T2D [23]. We were unable to breed a sufficient number of homozygous knockout mice due to high neonatal mortality resulting from respiratory failure, so the HFD cohort consisted of WT and heterozygous KDM5B-deficient mice.

**Figure 5:**
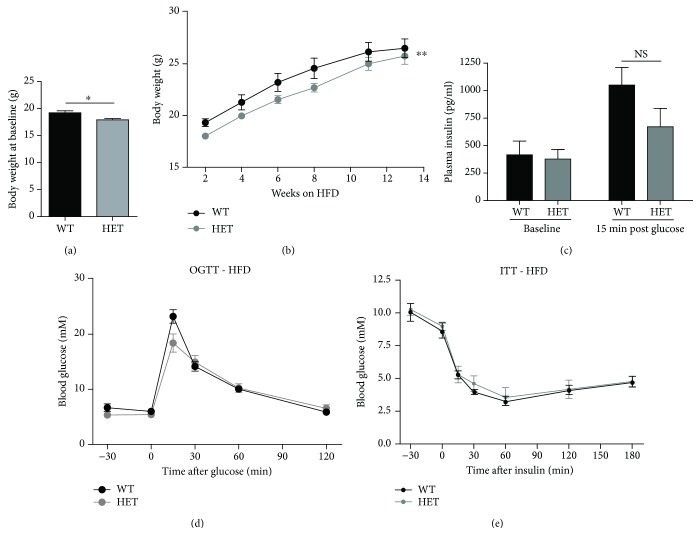
HFD cohort–phenotypic characterization. Wild-type (*n* = 9) and haploinsufficient mice (*n* = 7) were fed HFD (consisting of 60% fat compared to 11% in normal chow) for 13 weeks from age of 2-5 weeks. (a) Body weight at day 0 of HFD diet. (b) Body weight during 13 weeks of HFD. (c, d) OGTT. Following 13 weeks of HFD, mice were fasted overnight (16-18 hours) with access to water ad libitum. Blood samples were taken from a tail puncture at the indicated time points for measurement of glucose. (c) For measurement of insulin, a blood sample was taken from orbital sinus at time points -30 and 15 min for each genotype during OGTT. (d) Glucose excursion curves are shown for each genotype. AUC is shown for comparison of differences in glucose excursions. (e) ITT. Mice were fasted 2 hours prior to ITT with access to water ad libitum. Insulin (0.75 U/kg body weight) was injected i.p. Results are shown as means + SEMs. Statistical significance was determined using one- or two-way ANOVA. ^∗^ *p* < 0.05.

At baseline, the KDM5B-deficient mice exhibited decreased body weight (*p* < 0.05) (Figure 5(a)). They were equally susceptible to HFD-induced weight gain as WT mice and thus maintained a decreased body weight during 13 weeks of HFD (Figure 5(b)). The percentage of fat and lean mass was decreased and increased in KDM5B-deficient mice compared to WT mice, respectively (Supplementary Figures 4A-4B). The relative fat mass increased over time but was significantly decreased compared to WT mice (Supplementary Figure 4C), while there was no difference in the relative lean mass between KDM5B-deficient and WT mice following HFD (Supplementary Figure 4D).

We further investigated the impact of HFD on the metabolic phenotype of the mice. Following glucose challenge, there was no difference in insulin secretion of KDM5B-haploinsufficient mice compared to WT mice (Figure 5(c)), as we had observed in KDM5B-KO mice fed normal chow (Figure 3(b) and Figure 4(h)). The blood glucose levels of KDM5B-deficient mice were similar to those of WT mice during OGTT (Figure 5(d)), and there was no difference between WT and KDM5B-deficient mice when conducting ITT (Figure 5(e)). There was furthermore no difference in the levels of plasma GH or IGF-1 following HFD (Supplementary Figures 4E-4F).

## 4. Discussion

In this study, we wished to investigate the effect of lysine demethylase inhibition *in vivo* on experimental diabetes and elucidate the specific role of KDM5B on insulin secretion. While *in vivo* administration of the pharmacological inhibitor GSK-J4 failed to prevent the development of autoimmune diabetes, our findings suggest that KDM5B plays an important role in regulating somatic growth as well as maintaining glucose homeostasis *in vivo*.

KDM5B has been shown to be important for respiratory and neural developmental processes in mice [7, 19], but the role of KDM5B in beta-cell function has not previously been elucidated. Here, we show that KDM5B-KO resulted in enlarged islets of Langerhans with an increased fraction of beta-cell area compared to WT mice. Despite secreting less insulin than WT mice, KDM5B-KO mice were able to maintain normoglycemia following OGTT conducted at different ages. ITT showed indications of improved insulin sensitivity, and western blot analysis conducted in skeletal muscles revealed increased phosphorylation of proteins involved in the insulin signaling pathway suggesting an increased activity. KDM5B-KO mice furthermore displayed reduced somatic growth and alterations in body composition at different ages, associated with reduced GH action. When challenged with HFD, *Kdm5b*-deficient mice maintained the baseline reduction in body weight but were not protected against HFD-induced weight gain and responded similarly as WT mice regarding blood glucose levels.

We have previously shown that the KDM inhibitor GSK-J4 improves GSIS *in vitro* [9]. Here, we show GSK-J4 also potentiates insulin secretion *in vivo*, associated with increased pancreatic islet H3K4me3 levels. From GSEA, we show that GSK-J4 leads to significant upregulation of genes belonging to olfactory receptors and glutamate receptors. Interestingly, a recent study reveals that pancreatic islets and a beta-cell line exhibit the expression of olfactory receptors and that these receptors promote GSIS possibly via the phospholipase C-inositol triphosphate (PLC) pathway [24]. Furthermore, glutamate has been found to act as an excitatory neurotransmitter in the islets leading to improved insulin secretion [25, 26]. The mechanisms behind GSK-J4-mediated improved GSIS might thus involve increased expression of olfactory and glutamate receptors, leading to potentiated signaling for insulin secretion.

Methylation of H3K4 is important for the transcription of essential beta-cell genes [12, 27, 28], and preserved H3K4 methylation is associated with improved insulin secretion [13, 29]. We therefore hypothesized that preservation of H3K4 methylation achieved by KO of KDM5B would improve beta-cell function. In contrast to our expectations, GSIS conducted in islets from KDM5B-KO mice revealed reduced insulin secretion following the glucose challenge. Immunohistochemical analysis of pancreatic sections showed that KDM5B-KO mice exhibited a higher number of enlarged islets compared to WT mice.

Studies showing that knockdown or knockout of H3K4 methyltransferases leads to decreased expression of featured beta-cell genes and impaired GSIS via decreased H3K4 methylation have either been carried out in beta-cell lines, islets, or in mice with beta-cell-specific knockout [12, 13, 27, 28]. As our study was performed in a constitutive whole-body KDM5B-KO mouse, secondary pathology derived from the effects of the knockout on other cell types than islets most likely takes place, e.g., on the IGF-2 expression in the liver and many other tissues as detailed below. It is therefore possible that KDM5B-KO increases proliferation yielding large islets, with lower secretory capacity than small islets [30, 31] or nonproliferating beta-cells [32], thereby abrogating any positive effect of KDM5B-KO on insulin secretion. In accordance, it has been shown that the H3K4 methyltransferase SETD1A catalyzes methylation of heat-shock protein 70 (HSP70), leading to nuclear localization and growth promotion via direct interaction with Aurora kinase B [33]. Finally, it should be kept in mind that compensatory upregulation of other KDMs may confound the phenotype, e.g., KDM5A which regulates differentiation, cell cycle, and mitochondrial function, and KDM5C which controls neuronal function and viability.

Interestingly, we found that KDM5B-KO mice were able to compensate for the decreased insulin secretion by improving insulin sensitivity and thus maintained normoglycemia following OGTT. This finding is in accordance with studies showing that mice with deletion of the H3K4 methyltransferase Set7/9 display glucose intolerance [13], while mice deficient in another H3K4 methyltransferase myeloid-lineage leukemia (Mll2) display impaired glucose tolerance and insulin resistance [29].

Binding of insulin to its receptor initiates a signaling cascade resulting in glucose uptake in target tissues [34]. Following recruitment and phosphorylation of substrate molecules, e.g., insulin receptor substrate 1 and 2 (IRS1 and IRS2), activation of the downstream kinase serine/threonine protein kinase B (Akt/PKB) causes translocation of GLUT4 to the plasma membrane as well as phosphorylation and inactivation of glycogen synthase kinase-3beta (GSK-3beta) [35]. Inactivation of GSK-3beta removes the inhibitory phosphorylation of glycogen synthase (GS) thereby promoting synthesis of glycogen [36]. Several of these components have been shown to be regulated by lysine acetylation, as KDAC inhibitors leads to increased IRS2 expression, activity of IRS1 and Akt, and transcription of GLUT4 [37]. Our findings suggest that lysine demethylases play a similar regulatory role, as we found phosphorylation of Akt and GSK-3beta to be increased in the KDM5B-KO mouse. Such a regulatory role of lysine demethylases is supported by other studies [38–40].

The GH-IGF-1 axis is of particular importance for determining body length by stimulating linear bone growth [41, 42]. In accordance, we found the reduced body weight and length of KDM5B-KO mice to be associated with decreased GH and IGF-1 levels. Although we did not measure IGF-2 levels, it is possible that a compensatory upregulation of IGF-2 takes place. IGF-2 is mainly important for fetal growth, and does not compensate for reduced growth caused by GH and IGF-1 deficiencies in adult life [43]. However, since IGF-2 acts on the pancreatic beta-cells, an upregulation of IGF-2 would support beta-cell proliferation, which would explain the increased size and insulin-positive area as observed in islets from KDM5B-KO mice. In fact, IGF-2 is intricately regulated by imprinting and epigenetic control [44]. Deficiency of GH is furthermore associated with reduced growth, fat accumulation, and increased insulin sensitivity [45]. Interestingly, mice deficient in PICK1, a protein important for general vesicle biogenesis, are also deficient in GH [46] and display reduced growth, fat accumulation, and improved insulin sensitivity. As we observed similar phenotypical characteristics in KDM5B-KO mice, methylation may regulate transcription of GH or activity of the GH receptor signaling, as observed with other hormone receptors [47].

In summary, our findings reveal a novel role of KDM5B in body growth and metabolism, as KDM5B-KO reduced body weight and length and improved insulin sensitivity. The repressive effect of KDM5B on gene transcription, which is essential in order to avoid inappropriate gene expression during embryonic development [7], might have undesired effects later in life and inappropriately shut off the expression of genes involved in beneficial metabolic pathways. While the role of KDM5B in beta-cell function should be further examined, e.g., by creating a beta-cell-specific KO mouse, the beneficial effects of KDM5B-KO on insulin sensitivity should be assessed by investigating the expression and function of KDM5B in insulin-sensitive tissues.

## Data Availability

The data used to support the findings of this study are included within the article as well as within the supplementary information file.

## Supplementary Materials

Supplementary 1 Supplementary Figure 1. (A) Representative images of islet morphology. Pancreatic tissues were dissected from wild-type (WT) (*n* = 8), haploinsufficient (heterozygous, HET) (*n* = 6), and knockout (KO) (*n* = 3) mice, and islets were immunostained with either insulin (brown) or glucagon (blue) antibodies.

Supplementary 2 Supplementary Figure 2. (A) Insulin content measured ex vivo following GSIS (*n* = 3‐4). Results are shown as means + SEMs. Statistical significance was determined by unpaired *t*-test. (B-E) Absolute and relative lean and fat mass was determined in unanesthetized female mice (*n* = 5‐8) of age 7-11 weeks using a magnetic resonance imaging (MRI) scanner. Results are shown as means + SEMs. Statistical significance was determined by one-way ANOVA. ^∗^ *p* < 0.05, ^∗∗^ *p* < 0.01.

Supplementary 3 Supplementary Figure 3. (A-D) Absolute lean and fat mass was determined in unanesthetized female mice (*n* = 5‐8) of age 30-35 weeks using a MRI scanner. (E-G) Following sacrifice, weight of gonadal, subcutaneous, and retroperitoneal fat tissue was measured and normalized to body weight. (H) Protein expression levels of IR-*β* was determined by quantification of western blots on muscle samples from female mice (*n* = 4‐5) of age 37-42 weeks. Results are shown as means + SEMs. Statistical significance was determined by one-way ANOVA. ^∗∗^ *p* < 0.01.

Supplementary 4 Supplementary Figure 4. Wild-type (*n* = 9) and heterozygous mice (*n* = 7) were fed HFD for 13 weeks from the age of 2-5 weeks. (A-D) Absolute and relative fat and lean mass during 13 weeks of HFD. (E-F) GH and IGF-1 levels were measured in plasma from mice of age 23-26 weeks. Results are shown as means + SEMs. Statistical significance was determined using two-way ANOVA or unpaired *t*-test. ^∗∗^ *p* < 0.01.

Supplementary 5 Supplementary Table 1. Expression data from INS-1 832/13 cells in the absence or presence of the inhibitor GSK-J4. GSEA yielded two gene sets with upregulated expression.
