# Supplementary material for: The Lysine Demethylase KDM5B Regulates Islet Function and Glucose Homeostasis
Source: J Diabetes Res. 2019 Jul 28;2019:5451038. doi: 10.1155/2019/5451038 (PMC6701283; doi:10.1155/2019/5451038)
Supplement: Supplementary 1 — Supplementary Figure 1. (A) Representative images of islet morphology. Pancreatic tissues were dissected from wild-type (WT) (n = 8), haploinsufficient (heterozygous, HET) (n = 6), and knockout (KO) (n = 3) mice, and islets were immunostained with either insulin (brown) or glucagon (blue) antibodies. [file 5451038.f1.docx]

**A**


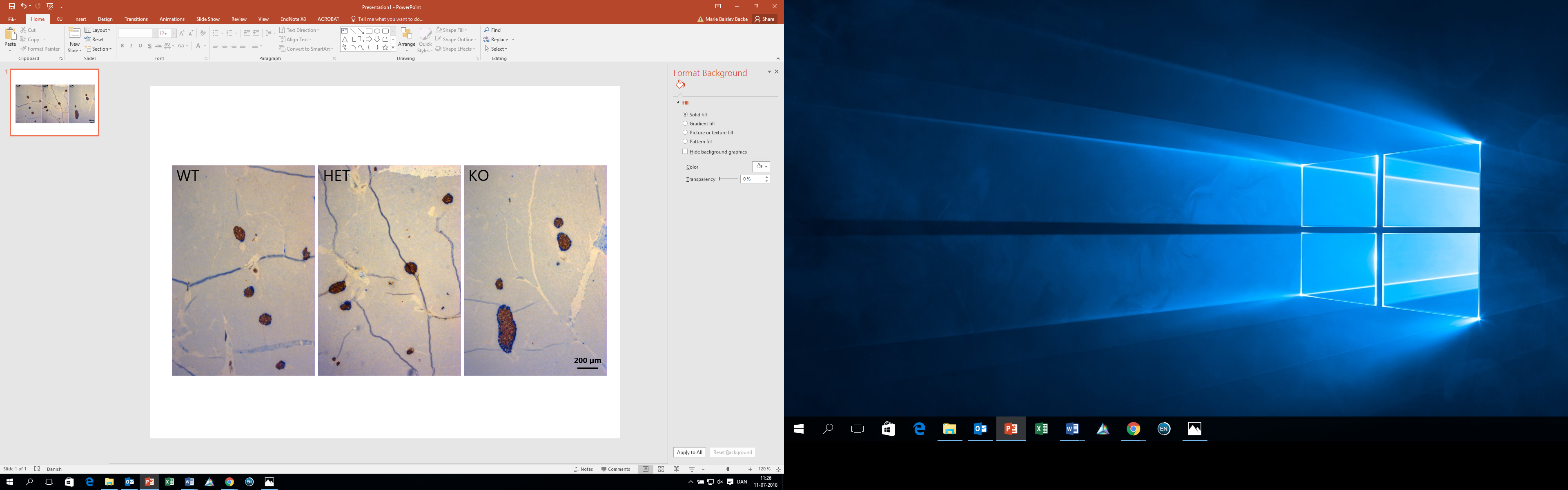


**Supplementary figure 1.** A) Representative images of islet morphology. Pancreatic tissues were dissected from wildtype (WT) (n=8), haploinsufficient (heterozygous, HET) (n=6) and knockout (KO) (n=3) mice and islets were immunostained with either insulin (brown) or glucagon (blue) antibodies.
