# Supplementary material for: The Lysine Demethylase KDM5B Regulates Islet Function and Glucose Homeostasis
Source: J Diabetes Res. 2019 Jul 28;2019:5451038. doi: 10.1155/2019/5451038 (PMC6701283; doi:10.1155/2019/5451038)
Supplement: Supplementary 2 — Supplementary Figure 2. (A) Insulin content measured ex vivo following GSIS (n = 3‐4). Results are shown as means + SEMs. Statistical significance was determined by unpaired t-test. (B-E) Absolute and relative lean and fat mass was determined in unanesthetized female mice (n = 5‐8) of age 7-11 weeks using a magnetic resonance imaging (MRI) scanner. Results are shown as means + SEMs. Statistical significance was determined by one-way ANOVA. ∗ p < 0.05, ∗∗ p < 0.01. [file 5451038.f2.docx]

**A B C**

**D E**

**Supplementary figure 2. A)** Insulin content measured ex vivo following GSIS (n=3-4). Results are shown as means + SEMs. Statistical significance was determined by unpaired t-test. **B-E)** Absolute and relative lean and fat mass was determined in unanesthetized female mice (n=5-8) of age 7-11 weeks of age using a Magnetic Resonance imaging (MRI) scanner. Results are shown as means + SEMs. Statistical significance was determined by one-way ANOVA. ** p<0.05, ** p<0.01.*
