# Supplementary material for: The Lysine Demethylase KDM5B Regulates Islet Function and Glucose Homeostasis
Source: J Diabetes Res. 2019 Jul 28;2019:5451038. doi: 10.1155/2019/5451038 (PMC6701283; doi:10.1155/2019/5451038)
Supplement: Supplementary 3 — Supplementary Figure 3. (A-D) Absolute lean and fat mass was determined in unanesthetized female mice (n = 5‐8) of age 30-35 weeks using a MRI scanner. (E-G) Following sacrifice, weight of gonadal, subcutaneous, and retroperitoneal fat tissue was measured and normalized to body weight. (H) Protein expression levels of IR-β was determined by quantification of western blots on muscle samples from female mice (n = 4‐5) of age 37-42 weeks. Results are shown as means + SEMs. Statistical significance was determined by one-way ANOVA. ∗∗ p < 0.01. [file 5451038.f3.docx]

**A B**

**C D**

**E F G**

**H**

**Supplementary figure 3. A-D)** Absolute lean and fat mass was determined in unanesthetized female mice (n=5-8) of age 30-35 weeks using a MRI scanner. **E-G)** Following sacrifice, weight of gonadal, subcutanoues and retroperitoneal fat tissue was measured and normalized to body weight. **H)** Protein expression levels of IR-β was determined by quantification of Western blots on muscle samples from female mice (n=4-5) of age 37-42 weeks. Results are shown as means + SEMs. Statistical significance was determined by one-way ANOVA. ** *p<0.01.*
