# Supplementary material for: The Lysine Demethylase KDM5B Regulates Islet Function and Glucose Homeostasis
Source: J Diabetes Res. 2019 Jul 28;2019:5451038. doi: 10.1155/2019/5451038 (PMC6701283; doi:10.1155/2019/5451038)
Supplement: Supplementary 4 — Supplementary Figure 4. Wild-type (n = 9) and heterozygous mice (n = 7) were fed HFD for 13 weeks from the age of 2-5 weeks. (A-D) Absolute and relative fat and lean mass during 13 weeks of HFD. (E-F) GH and IGF-1 levels were measured in plasma from mice of age 23-26 weeks. Results are shown as means + SEMs. Statistical significance was determined using two-way ANOVA or unpaired t-test. ∗∗ p < 0.01. [file 5451038.f4.docx]

**A B**

**C D**

**E F**

**Supplementary figure 4.** Wild type (n=9) and heterozygous mice (n=7) were fed HFD for 13 weeks from age of 2-5 weeks. **A-D)** Absolute and relative fat and lean mass during 13 weeks of HFD. **E-F)** GH and IGF-1 levels were measured in plasma from mice of age 23-26 weeks. Results are shown as means + SEMs. Statistical significance was determined using two-way ANOVA or unpaired t-test. ** *p<0.01.*
