# Supplementary material for: The Lysine Demethylase KDM5B Regulates Islet Function and Glucose Homeostasis
Source: J Diabetes Res. 2019 Jul 28;2019:5451038. doi: 10.1155/2019/5451038 (PMC6701283; doi:10.1155/2019/5451038)
Supplement: Supplementary 5 — Supplementary Table 1. Expression data from INS-1 832/13 cells in the absence or presence of the inhibitor GSK-J4. GSEA yielded two gene sets with upregulated expression. [file 5451038.f5.docx]

| **Gene set ID and name**  (Pathway group) | **Genes contributing to the enrichment score** | **Regulated genes/total** | **ES** | ***p* value** | ***q* value** |
| --- | --- | --- | --- | --- | --- |
| **RNO04740**  **Olfactory transduction**  (Sensory system) | *Or5ap2, Or5m8, Or51f2, Or56a3, Or2z1, Or5a1, Or2ag1, Or4s2, Or52e6, Or2a25, Or2d2, Or4k2, Or10r2, Or6n1, Or51i1, Or5k2, Or2l13, Or52n1, Or13j1, Or51b2, Or2ag2, Or13a1, Or7g3, Or10a4, Or52e4, Or4l1, Or2t1, Or51a7, Or6t1, Or51b6, Or2j3, Or10g2, Or2b2, Or4f6, Guca1a, Or52a1, Or1s1, Or6c68, Or56b4, Or5b3, Or11a1, Or10x1, Or52a5, Or13g1, Or4d11, Or2h1, Or10t2, Or4f15, Or1d2, Or9i1, Or52b4, Or5b21, Or9q1, Or13c3, Or1i1, Or4d1, Or7a5, Clca2, Or1g1, Or2b11, Or1m1, Or52d1, Or2v2, Or1n1, Or7d4, Or51i2, Or8a1, Or1k1, Or4k15, Cnga3, Or8j3, Or51e1, Or4d9, Or2h2, Or51a2, Or4c12, Or10g4, Or6k3, Or4f5, Or5c1, Or4d6, Or1a1, Or10ag1, Or4a47, Or10k2, Or10h2, Or2w3, Or52j3, Or11g2, Or51g2, Or6c3, Or4a15, Or6q1, Or4c11, Or6c65, Arrb2, Prkacb, Or52l1, Or6b1, Or2l3, Or8g5, Or5d13, Or4k1, Or5ac2, Or6k2, Or2a2, Or4d10, Or4c16, Or5m1, Pdc, Or5b12.* | 111/264 | 0.24 | 0.000 | 0.000 |
| **RNO04080 Neuroactive ligand-receptor interaction**  (Signaling molecules and interaction) | *Gria3, Hrh4, Chrna1, Prl, Grm3, Fpr1, P2rx1, Taar1, Calcrl, Htr7, Taar6, Fshr, Cysltr1, Glrb, Glp2r, Mc2r, P2ry13, Htr5a, Gabra6, Gpr156, Grm1, Sstr4, Tbxa2r, Lpar3, Agtr2, Htr2b, Chrnd, Glra2, Adra1b, Htr4, Oprl1, Cnr1, Rxfp2, Bdkrb2, Mtnr1b, Htr1a, Nmur1, Htr1d, Sstr3, Prlhr, Cnr2, Npy2r, Grik3, S1pr4, Htr2a, Adra1a, Grin2b, Trhr, Gabrp, Gabre, Gabrd, Drd3, S1pr3, F2rl2, Chrm5, Gabra1, Gpr83, Avpr2, Gabrg1, Nmur2, Grik5, P2ry6, Chrna5, Ltb4r2, Npy5r, Gipr, Gpr50, Gabra5, Adora1, Htr2c, Tacr1, Grm6, Sstr1, Drd1, Gabbr2, Hrh3, Adora3, Lpar6, Trpv1, Adora2a, Gcgr, P2rx4, Grm2, Ppyr1, F2r, Grin2c, Chrna3.* | 87/241 | 0.16 | 0.000 | 0.000 |

**Supplementary table 1.** Expression data from INS-1 832/13 cells in absence or presence of the inhibitor GSK-J4. GSEA yielded two gene sets with upregulated expression.
